# Supplementary material for: Knowledge, attitude, and practice toward perioperative neurocognitive disorders among healthcare workers in Shandong, China: a cross-sectional study
Source: PeerJ. 2025 Dec 9;13:e20450. doi: 10.7717/peerj.20450 (PMC12700114; doi:10.7717/peerj.20450)
Supplement: Supplemental Information 1 [file peerj-13-20450-s001.docx]

Supplementary Table 1. Correctness of knowledge

| Knowledge | Correct rate, n (%) |
| --- | --- |
| 1. PND includes postoperative delirium, delayed neurocognitive recovery, postoperative neurocognitive disorder, and both mild and major cognitive impairments. | 275(89.0) |
| 2. Postoperative delirium is the earliest and most prominent form of PND. | 250(80.9) |
| 3. PND often manifests with symptoms including acute mental confusion, hallucinations, disorientation, inappropriate behavior, language impairments, and transient memory loss. | 264(85.4) |
| 4. The pathogenesis of PND may be associated with the brain's response to neuroinflammation and oxidative stress induced by surgery. | 246(79.6) |
| 5. Advanced age is an important risk factor for PND | 269(87.1) |
| 6. Elderly patients should avoid using anticholinergics and benzodiazepines preoperatively. | 242(78.3) |
| 7. The type of surgery is not associated with the occurrence of PND. (False) | 196(63.4) |
| 8. Currently, neuropsychological testing is regarded as the "gold standard" for diagnosing PND in clinical practice. | 161(52.1) |
| 9. PND is postoperative delirium. (False) | 186(60.2) |
| 10. The implementation of enhanced recovery after surgery_ERAS_protocols may reduce the incidence of PND. | 259(83.8) |
| 11. PND patients can be treated by providing cognitive stimulation, enhancing circadian rhythms, and using opioids for analgesia. | 217(70.2) |
| 12. The occurrence of PND will compromise patient autonomy and reduce quality of life, extend hospital stays, and increase morbidity and mortality rates | 259(83.8) |
