# Supplementary material for: Knowledge, attitude, and practice toward perioperative neurocognitive disorders among healthcare workers in Shandong, China: a cross-sectional study
Source: PeerJ. 2025 Dec 9;13:e20450. doi: 10.7717/peerj.20450 (PMC12700114; doi:10.7717/peerj.20450)
Supplement: Supplemental Information 2 [file peerj-13-20450-s002.docx]

Supplementary Table 2. Attitudes of healthcare workers regarding PND, n (%).

| Attitude | Strongly agree | Agree | Neutral | Disagree | Strongly disagree |
| --- | --- | --- | --- | --- | --- |
| 1. PND requires the attention of healthcare professionals. | 257(83.17) | 43(13.92) | 8(2.59) | 1(0.32) | 0 |
| 2. It is necessary to enhance the awareness of PND among patients and their families and improve their cooperation. | 252(81.55) | 48(15.53) | 8(2.59) | 0 | 1(0.32) |
| 3. Medical staff need to fully grasp the relevant knowledge of PND. | 244(78.96) | 55(17.80) | 9(2.91) | 0 | 1(0.32) |
| 4. The prevention of PND primarily relies on anesthesiologists, rather than attending physicians and nurses. | 60(19.42) | 25(8.09) | 28(9.06) | 111(35.92) | 85(27.51) |
| 5. Strengthening the management of PND patients is irrelevant to the prevention and recovery of PND. | 58(18.77) | 16(5.18) | 11(3.56) | 96(31.07) | 128(41.42) |
| 6. Medical staff need to work as a team to assess the high-risk factors for PND occurrence and jointly prevent the onset of PND. | 247(79.94) | 50(16.18) | 8(2.59) | 3(0.97) | 1(0.32) |
| 7. I am willing to receive training on PND-related knowledge. | 221(71.52) | 73(23.62) | 13(4.21) | 2(0.65) | 0 |
