# Supplementary material for: Knowledge, attitude, and practice toward perioperative neurocognitive disorders among healthcare workers in Shandong, China: a cross-sectional study
Source: PeerJ. 2025 Dec 9;13:e20450. doi: 10.7717/peerj.20450 (PMC12700114; doi:10.7717/peerj.20450)
Supplement: Supplemental Information 3 [file peerj-13-20450-s003.docx]

Supplementary Table 3. Distribution of practice (%)

| Practice | Always | Often | Sometimes | Occasionally | Never |
| --- | --- | --- | --- | --- | --- |
| 1. I will inform the patient and their family about the risk of PND occurrence and the preventive measures. | 132(42.72) | 91(29.45) | 44(14.24) | 26(8.41) | 16(5.18) |
| 2. I would recommend that elderly patients undergo cognitive function training before surgery. | 121(39.16) | 58(18.77) | 66(21.36) | 30(9.71) | 34(11.00) |
| 3. I will inform the patient about the risks associated with PND. | 132(42.72) | 82(26.54) | 52(16.83) | 23(7.44) | 20(6.47) |
| 4. I will inquire about the patient's previous cognitive function. | 142(45.95) | 85(27.51) | 50(16.18) | 25(8.09) | 7(2.27) |
| 5. I would advise the patient to ensure adequate sleep. | 185(59.87) | 78(25.24) | 36(11.65) | 7(2.27) | 3(0.97) |
| 6. I would recommend non-opioid analgesics to help the patient manage pain. | 98(31.72) | 77(24.92) | 96(31.07) | 19(6.15) | 19(6.15) |
| 7. I will monitor the patient's brain and cognitive functions postoperatively. | 146(47.25) | 91(29.45) | 54(17.48) | 10(3.24) | 8(2.59) |
| 8. When a patient has high-risk factors for developing PND, I will actively take measures to prevent the occurrence of PND. | 167(54.05) | 85(27.51) | 39(12.62) | 12(3.88) | 6(1.94) |
| 9. I will actively participate in the professional training courses of PND. | 144(46.60) | 76(24.60) | 54(17.48) | 20(6.47) | 15(4.85) |
