# Supplementary material for: Knowledge, attitude, and practice toward perioperative neurocognitive disorders among healthcare workers in Shandong, China: a cross-sectional study
Source: PeerJ. 2025 Dec 9;13:e20450. doi: 10.7717/peerj.20450 (PMC12700114; doi:10.7717/peerj.20450)
Supplement: Supplemental Information 4 [file peerj-13-20450-s004.docx]

Table S4: The results of multiple comparisons of knowledge scores

| Variables | | MD | SE | Pvalue |
| --- | --- | --- | --- | --- |
| **Age** | |  |  |  |
| <30 | [30,40] | -0.625 | 0.430 | 0.443 |
| <30 | >40 | 0.418 | 0.425 | 0.979 |
| [30,40] | >40 | 1.042 | 0.363 | 0.013 |
| **Department** | |  |  |  |
| Surgery | Anesthesiology and operating room nurse | -0.652 | 0.359 | 0.210 |
| Surgery | The relevant internal medicine | 1.280 | 0.479 | 0.024 |
| Anesthesiology and operating room nurse | The relevant internal medicine | 1.932 | 0.441 | <0.001 |
