# Supplementary material for: Knowledge, attitude, and practice toward perioperative neurocognitive disorders among healthcare workers in Shandong, China: a cross-sectional study
Source: PeerJ. 2025 Dec 9;13:e20450. doi: 10.7717/peerj.20450 (PMC12700114; doi:10.7717/peerj.20450)
Supplement: Supplemental Information 5 [file peerj-13-20450-s005.docx]

Table S5: The results of multiple comparisons of attitude scores

| Variables | | MD | SE | Pvalue |
| --- | --- | --- | --- | --- |
| **Age** | |  |  |  |
| <30 | [30,40] | -2.011 | 0.572 | 0.001 |
| <30 | >40 | -1.525 | 0.564 | 0.022 |
| [30,40] | >40 | 0.486 | 0.482 | 0.944 |
| **Education** |  |  |  |  |
| Associated Degree and lower | Bachelor’s Degree | -1.932 | 0.842 | 0.067 |
| Associated Degree and lower | Master’s Degree and higher | -2.851 | 0.908 | 0.006 |
| Bachelor’s Degree | Master’s Degree and higher | -0.919 | 0.500 | 0.202 |
| **Professional title** |  |  |  |  |
| Junior and below | Intermediate | -0.900 | 0.521 | 0.255 |
| Junior and below | Vice senior and above | -1.700 | 0.536 | 0.005 |
| Intermediate | Vice senior and above | -0.799 | 0.517 | 0.369 |
| **Department** | |  |  |  |
| Surgery | Anesthesiology and operating room nurse | -0.652 | 0.359 | 0.210 |
| Surgery | The relevant internal medicine | 1.280 | 0.479 | 0.024 |
| Anesthesiology and operating room nurse | The relevant internal medicine | 1.932 | 0.441 | <0.001 |
