# Supplementary material for: Knowledge, attitude, and practice toward perioperative neurocognitive disorders among healthcare workers in Shandong, China: a cross-sectional study
Source: PeerJ. 2025 Dec 9;13:e20450. doi: 10.7717/peerj.20450 (PMC12700114; doi:10.7717/peerj.20450)
Supplement: Supplemental Information 6 [file peerj-13-20450-s006.docx]

Table S6. Cronbach's Alpha

|  | Reference | Results |
| --- | --- | --- |
| Entire questionnaire | >0.80 Good | 0.873 |
| Knowledge dimension | >0.80 Good | 0.818 |
| Attitude dimension | >0.80 Good | 0.694 |
| Practice dimension | >0.80 Good | 0.917 |
