# Supplementary material for: Knowledge, attitude, and practice toward perioperative neurocognitive disorders among healthcare workers in Shandong, China: a cross-sectional study
Source: PeerJ. 2025 Dec 9;13:e20450. doi: 10.7717/peerj.20450 (PMC12700114; doi:10.7717/peerj.20450)
Supplement: Supplemental Information 7 [file peerj-13-20450-s007.docx]

Questionnaire No.

Dear Participant,

We are researchers from Jinan Central Hospital (Central Hospital Affiliated to Shandong First Medical University), and we sincerely invite you to participate in our research project. This study aims to understand healthcare professionals' knowledge, attitudes, and willingness to practice regarding perioperative neurocognitive disorders (PND) to provide a basis for developing scientific intervention strategies that may help more people in the future and improve their health conditions. Your participation in this study is voluntary. If you agree to participate, please refer to the following instructions.

First, please complete the questionnaire. There are no right or wrong answers between the dimensions of attitude and practice; you only need to fill it out based on your actual situation. You may raise any questions during the answering process, and please submit them promptly upon completion. It is worth noting that some items in the 'Practice' dimension of this questionnaire are mainly applicable to elective surgery scenarios.

Second, this study involves a simple questionnaire survey that will not cause any harm to your physical or psychological well-being. However, it will involve some privacy issues, such as your gender and age. We assure you that your information will be strictly confidential and will not be disclosed. Please feel free to fill it out.

Third, as a participant, you are entitled to access information related to this study and its progress at any time. If you decide to withdraw from the study, please inform us, and your data will not be included in the research findings.

Finally, we sincerely thank you for taking the time out of your busy schedule to support our scientific research!

**Part I Basic Information**

| **1. Do you agree to participate in this survey research? (Consent to this survey is equivalent to signing an informed consent form.)** | I have acknowledged and agreed that the collected data will be used for scientific research. |
| --- | --- |

| **2.Your age:** | Years old |
| --- | --- |
| **3.Your gender:** | a.Male  b.Female |
| **4.Your residence:** | a.Rural  b.Urban  c.Suburb |
| **5.Your education:** | a.Junior college and below  b.Undergraduate  c.Postgraduate and above |
| **6.Your type of occupation:** | a.Doctor  b.Nurse  c.Other: |
| **7. Your professional title** | a.Junior  b.Intermediate  c. Vice senior  d.Senior  e.No title |
| **8. The department you work in is** | a. Neurosurgery  b. Cardiac Surgery  c. Orthopedic Surgery  d. Urology  e. General Surgery  f. Hepatobiliary Surgery  g. Vascular Surgery  h. Anesthesiology  i. Neurology  j. Cardiology  k. Gastroenterology  l. Department of operating room  m. Others. |
| **9. Duration of your work experience:** | Years |
| **10. The type of hospital you are working in is** | a.Public primary  b.Public secondary  c.Public tertiary  d. Specialist hospital  e. Private medical institution |
| **11. Have you ever participated in the training of PND:** | a.Yes  b.No  c.Unclear |

**Part II-Knowledge**

Please select " Correct" or "Wrong" depending on your comprehension of the question. If you are unsure of the answer to a question, please select “Unsure”.

| **1. PND includes postoperative delirium, delayed neurocognitive recovery, postoperative neuro-cognitive disorder, and both mild and major cognitive impairments.** | **a.Correct** | **b.Wrong** | **c.Unsure** |
| --- | --- | --- | --- |
| **2. Postoperative delirium is the earliest and most prominent form of PND.** | **a.Correct** | **b.Wrong** | **c.Unsure** |
| **3. PND often manifests with symptoms including acute mental confusion, hallucinations, disorientation, inappropriate behavior, language impairments, and transient memory loss.** | **a.Correct** | **b.Wrong** | **c.Unsure** |
| **4. The pathogenesis of PND may be associated with the brain's response to neuroinflammation and oxidative stress induced by surgery.** | **a.Correct** | **b.Wrong** | **c.Unsure** |
| **5. Advanced age is an important risk factor for PND** | **a.Correct** | **b.Wrong** | **c.Unsure** |
| **6. Elderly patients should avoid using anticholinergic and benzodiazepines preoperatively.** | **a.Correct** | **b.Wrong** | **c.Unsure** |
| **7. The type of surgery is not associated with the occurrence of PND. (False)** | **a.Correct** | **b.Wrong** | **c.Unsure** |
| **It is recommended that patients with hyperuricemia consume more dairy products and fresh vegetables, drink moderate amounts of water, and limit their intake of soy products.** | **a.Correct** | **b.Wrong** | **c.Unsure** |
| **8. Currently, neuropsychological testing is regarded as the "gold standard" for diagnosing PND in clinical practice.** | **a.Correct** | **b.Wrong** | **c.Unsure** |
| **9. PND is postoperative delirium. (False)** | **a.Correct** | **b.Wrong** | **c.Unsure** |
| **10. The implementation of enhanced recovery after surgery_ERAS_protocols may reduce the incidence of PND.** | **a.Correct** | **b.Wrong** | **c.Unsure** |
| **11. PND patients can be treated by providing cognitive stimulation, enhancing circadian rhythms, and using opioids for analgesia.** | **a.Correct** | **b.Wrong** | **c.Unsure** |
| **12. The occurrence of PND will compromise patient autonomy and reduce quality of life, extend hospital stays, and increase morbidity and mortality rates** | **a.Correct** | **b.Wrong** | **c.Unsure** |

**Part III-Attitude**

Please choose from “Strongly agree” to “Strongly disagree” depending on whether you agree with the description in the question.

| **1. PND requires the attention of healthcare professionals.** | **a.Strongly agree** | **b.Agree** | **c.Neutral** | **d.Disagree** | **e.Strongly disagree** |
| --- | --- | --- | --- | --- | --- |
| **2. It is necessary to enhance the awareness of PND among patients and their families and improve their cooperation.** | **a.Strongly agree** | **b.Agree** | **c.Neutral** | **d.Disagree** | **e.Strongly disagree** |
| **3. Medical staff need to fully grasp the relevant knowledge of PND.** | **a.Strongly agree** | **b.Agree** | **c.Neutral** | **d.Disagree** | **e.Strongly disagree** |
| **4. The prevention of PND primarily relies on anesthesiologists, rather than attending physicians and nurses.** | **a.Strongly agree** | **b.Agree** | **c.Neutral** | **d.Disagree** | **e.Strongly disagree** |
| **5. Strengthening the management of PND patients is irrelevant to the prevention and recovery of PND.** | **a.Strongly agree** | **b.Agree** | **c.Neutral** | **d.Disagree** | **e.Strongly disagree** |
| **6. Medical staff need to work as a team to assess the high-risk factors for PND occurrence and jointly prevent the onset of PND.** | **a.Strongly agree** | **b.Agree** | **c.Neutral** | **d.Disagree** | **e.Strongly disagree** |
| **7. I am willing to receive training on PND-related knowledge.** | **a.Strongly agree** | **b.Agree** | **c.Neutral** | **d.Disagree** | **e.Strongly disagree** |

**Part IV-Practice**

Please choose from “Always” to “Never” depending on whether you agree with the description in the question.

| **1. I will inform the patient and their family about the risk of PND occurrence and the preventive measures.** | **a.Always** | **b.Usually** | **c.Neutral** | **d.Occasionally** | **e.Never** |
| --- | --- | --- | --- | --- | --- |
| **2. I would recommend that elderly patients undergo cognitive function training before surgery.** | **a.Always** | **b.Usually** | **c.Neutral** | **d.Occasionally** | **e.Never** |
| **3. I will inform the patient about the risks associated with PND.** | **a.Always** | **b.Usually** | **c.Neutral** | **d.Occasionally** | **e.Never** |
| **4. I will inquire about the patient's previous cognitive function.** | **a.Always** | **b.Usually** | **c.Neutral** | **d.Occasionally** | **e.Never** |
| **5. I would advise the patient to ensure adequate sleep.** | **a.Always** | **b.Usually** | **c.Neutral** | **d.Occasionally** | **e.Never** |
| **6. I would recommend non-opioid analgesics to help the patient manage pain.** | **a.Always** | **b.Usually** | **c.Neutral** | **d.Occasionally** | **e.Never** |
| **7. I will monitor the patient's brain and cognitive functions postoperatively.** | **a.Always** | **b.Usually** | **c.Neutral** | **d.Occasionally** | **e.Never** |
| **8. When a patient has high-risk factors for developing PND, I will actively take measures to prevent the occurrence of PND.** | **a.Always** | **b.Usually** | **c.Neutral** | **d.Occasionally** | **e.Never** |
| **9. I will actively participate in the professional training courses of PND.** | **a.Always** | **b.Usually** | **c.Neutral** | **d.Occasionally** | **e.Never** |
