# Supplementary material for: Knowledge, attitude, and practice toward perioperative neurocognitive disorders among healthcare workers in Shandong, China: a cross-sectional study
Source: PeerJ. 2025 Dec 9;13:e20450. doi: 10.7717/peerj.20450 (PMC12700114; doi:10.7717/peerj.20450)
Supplement: Supplemental Information 10 [file peerj-13-20450-s010.doc]

| **non-English text** | **translations for the non-English text** |
| --- | --- |
| 整理后（去除重复和小于90） - 副本 - 副本 (2) | **Data after the application of inclusion criteria** |
